# Behavioral and Psychosocial Effects of Participant-Visible Continuous Glucose Monitoring in Adults Without Diabetes: A Systematic Review

**DOI:** 10.3390/medicina62091814

**Published:** 2026-09-20

**Authors:** Hima Bindu Makkena, Yasaswini Nimmagadda, Navya Boinapelly, Anju George, Nagasravani Daruvuri, Ramesh Raj Sunar, Srinayani Guda

**Affiliations:** 1 Katuri Medical College & Hospital, Guntur 522019, Andhra Pradesh, India; 2 Department of Internal Medicine, Malla Reddy Institute of Medical Sciences, Hyderabad 500055, Telangana, India; nimmagaddayasaswini@gmail.com; 3 Mamata Medical College, Khammam 507002, Telangana, India; navyarao.b@gmail.com; 4 Department of Internal Medicine, Amala Institute of Medical Sciences, Thrissur 680555, Kerala, India; anjugeorge47656@gmail.com; 5 Kasturba Medical College, Manipal 576104, Karnataka, India; sravani.d.1004@gmail.com; 6 Internal Medicine, Al Hana Modern Medical Center, Dubai P.O. Box 24824, United Arab Emirates; r.sunarmd@gmail.com; 7 Department of Internal Medicine, Hackensack Meridian Health Palisades Medical Center, North Bergen, NJ 07047, USA; srinayani.guda1@hmhn.org

**Keywords:** continuous glucose monitoring, adults without diabetes, dietary behavior, physical activity, psychosocial, well-being, digital health, wearable technology, systematic review

## Abstract

*Background and Objectives*: Continuous glucose monitoring (CGM) is increasingly used beyond diabetes care, but whether participant-visible feedback changes behavior or psychosocial outcomes in adults without diabetes remains uncertain. We evaluated dietary behavior/adherence, physical activity, psychosocial outcomes, and device burden. *Materials and Methods*: This PROSPERO-registered systematic review (CRD420261356163) searched seven bibliographic databases and two trial registries, followed by citation searching. Randomized and nonrandomized intervention studies and qualitative/mixed-methods evidence were eligible. RoB 2, ROBINS-I, MMAT 2018, and GRADE were applied. Clinical and methodological heterogeneity precluded meta-analysis. *Results*: Searches yielded 6265 database/registry records; 2627 unique records were screened. Citation searching added 431 new records and two eligible studies, giving 12 studies. Two randomized dietary comparisons suggested selected improvements; one 8-week trial comparing CGM plus low-glycemic-index/load education with education alone reported between-group reductions in carbohydrate intake (−15.2 g; 95% CI −20.9 to −9.5), glycemic index (−10.0; 95% CI −13.1 to −6.9), and glycemic load (−19.9; 95% CI −29.0 to −10.7). Physical activity showed no clear difference (−28.5 MET-min/week; 95% CI −171.9 to 114.8). Psychosocial evidence was insufficient to establish benefit or exclude harm; device burden evidence was sparse. Certainty was low for dietary outcomes and very low for the other domains. *Conclusions*: Participant-visible CGM may support selected dietary changes as a biofeedback adjunct within structured education or lifestyle interventions, but large, durable benefits and stand-alone efficacy are not established.

## 1. Introduction

Continuous glucose monitoring was developed to support day-to-day glucose management in people with diabetes, but the technology has moved rapidly into wellness, sports, and consumer health settings. In people without diabetes, the appeal is less about avoiding acute dysglycemia and more about making otherwise invisible postprandial glucose responses visible in real time. This feedback may encourage experimentation with food choice, meal timing, exercise, sleep, and other daily behaviors. Reviews and commentaries have therefore proposed CGM as a tool for personalized nutrition and self-regulation outside traditional diabetes care [1,2].

The same feedback loop also creates reasons for caution. Transient postprandial glucose rises can occur in people with normal glucose tolerance [1]. Such excursions should not, in isolation, be equated with clinical dysglycemia, and their significance requires appropriate clinical interpretation [1,3]. Without this distinction, users may interpret physiological variation as disease, potentially encouraging unnecessary dietary restriction or anxiety [3]. A recent narrative review highlighted the limited quality of evidence for CGM use in people not living with diabetes and specifically identified device acceptability and possible adverse effects on eating behavior as under-studied areas [3].

A broader 2026 systematic review of 23 studies in non-diabetic populations evaluated glycemic outcomes, body weight, dietary behavior, adherence, and measurement performance [4]. That review is important context, but it addresses a wider clinical question than the present review. For behavior change claims, a central methodological issue is attribution: a study in which CGM is blinded, used only to measure physiology, or delivered together with intensive coaching cannot automatically establish that participant-visible glucose feedback itself produced the observed behavior. Likewise, changes in well-being during a dietary intervention cannot be treated as a CGM effect if CGM is present in every condition.

We therefore focused on participant-visible CGM and asked a narrower question: whether seeing glucose feedback was followed by a behavioral or psychosocial response. Primary outcomes were dietary behavior, dietary adherence, and physical activity; secondary outcomes were well-being, mood, anxiety, distress, and device-related psychological burden. Qualitative evidence and practical device harms were also retained because they are directly relevant to consumer use. Our objective was to determine what can currently be concluded while keeping randomized CGM-specific evidence separate from supportive multicomponent or uncontrolled evidence and from qualitative or descriptive experience.

## 2. Materials and Methods

### 2.1. Protocol and Reporting Framework

The review was prospectively registered in PROSPERO (CRD420261356163). The protocol specified a systematic review with conditional meta-analysis: pooling would be attempted only when population, intervention, outcome construct, measurement, and reporting were sufficiently comparable. Reporting followed PRISMA 2020 [5], and the final flow diagram was produced with the PRISMA2020 R package and Shiny application [6]. Because the quantitative evidence did not meet the conditions for meta-analysis, the synthesis was organized in accordance with SWiM principles [7].

### 2.2. Eligibility Criteria

Population: Adults older than 18 years without diabetes were eligible. The term “adults without diabetes” describes eligibility with respect to diabetes status and does not imply that all participants had objectively confirmed normal glucose tolerance. Type 1 diabetes, type 2 diabetes, gestational diabetes, clinically managed prediabetes, and pediatric and adolescent populations were excluded. Overweight or obesity alone was not an exclusion. Mixed populations were eligible only when an eligible non-diabetic subgroup was separately identifiable for the outcome of interest.

Interventions: Eligible studies evaluated active CGM in a free-living or real-world setting in which participants could access glucose data and where feedback was capable of prompting a behavioral or psychosocial response. Studies that used blinded CGM solely as a physiological measurement instrument were not treated as evidence of a CGM-caused behavioral effect.

Comparator and design: Depending on study design, comparators included no CGM feedback, baseline/pre-CGM status, or the same lifestyle intervention without CGM feedback. Randomized controlled trials and nonrandomized intervention studies, including cohort and pre–post designs, were eligible. Mixed-methods and qualitative evidence was retained when it directly described participant experience with visible CGM.

Outcomes: Primary behavioral outcomes were dietary intake/behavior, dietary adherence, and physical activity. Secondary psychosocial outcomes included psychological well-being, mood, anxiety, distress, and device-related distress. Practical harms and burdens, including sensor problems, scanning requirements, skin symptoms, adhesion difficulties, and device-related inconvenience, were also synthesized.

### 2.3. Information Sources and Search Strategy

The search covered PubMed/MEDLINE, Web of Science Core Collection, Scopus, Europe PMC, the Cochrane Central Register of Controlled Trials (CENTRAL), CINAHL Complete, and PsycINFO. ClinicalTrials.gov and the World Health Organization International Clinical Trials Registry Platform (WHO ICTRP) were searched for ongoing, unpublished, or linked studies. The bibliographic database searches were run on 15 April 2026 and covered records from 1 January 2015 up to the date of searching, using source-specific date filters where applicable. The 2015 start date was specified in the review protocol to focus on the period of increasing availability of modern CGM systems suitable for participant-visible glucose feedback in free-living settings. No language or study design restriction was applied.

Searches combined two concepts—continuous glucose monitoring AND healthy/non-diabetic population—using controlled vocabulary where available and title/abstract or equivalent free-text fields. Behavioral and psychosocial outcome terms were intentionally omitted because requiring an outcome term in the title or abstract could miss studies in which behavior was a secondary or qualitative outcome. The complete database-specific strategies, field mappings, export details, and retrieval counts are provided in Supplementary File S1.

The final retrieval counts were as follows: PubMed/MEDLINE 1167; Web of Science 1214; Scopus 1566; Europe PMC 1071; CENTRAL 633; CINAHL Complete 297; and PsycINFO 45 (5993 database records). ClinicalTrials.gov contributed 253 records and WHO ICTRP 19 records (272 registry records).

### 2.4. Study Selection, Report Linkage, and Citation Searching

After deduplication, records were screened against the prespecified eligibility criteria. Hima Bindu Makkena (HBM) performed the primary title/abstract screening, and each record received an independent second assessment by one of Yasaswini Nimmagadda, Navya Boinapelly, Anju George, Nagasravani Daruvuri, Ramesh Raj Sunar, or Srinayani Guda, with the workload distributed across reviewers. Potentially eligible reports were retrieved and assessed using the full article when available or the most complete available report when this was not. Report-level eligibility decisions and exclusion reasons were independently checked by a second reviewer, with disagreements resolved through discussion and consensus. Registry entries, protocols, preprints, duplicate representations, and related reports were linked to the underlying study so that multiple records from the same study were not counted as independent evidence. Each excluded report was assigned one primary reason for exclusion.

Supplementary citation searching was undertaken after the original database/registry screening. Reference lists of the ten initially included reports were checked first. When this backward search identified a new eligible study, that report became the sole seed for a second backward iteration; the second iteration yielded no further eligible study. Forward citation searching was then conducted from the 11-study set. Open citation indexes were queried, records already present in the original or backward-search universes were removed, and only genuinely new records were screened. HBM conducted the citation searching and primary eligibility screening of citation search records; the same independent second screening process was applied to the 431 genuinely new records, with the workload distributed among the other review team members. The new forward-search inclusion was used as the sole seed for a second forward iteration, which returned no citing records. The stopping rule was therefore met in both directions. Citation searching was performed in July 2026 using the same eligibility criteria as the original review.

### 2.5. Data Extraction, Source Verification, and Outcome Attribution

Study characteristics, intervention details, participant eligibility, behavioral and psychosocial outcomes, numerical results, source location, and CGM attribution were extracted into a standardized framework. During revision, we additionally documented the reported methods and thresholds used to assess diabetes or glucose tolerance status in each study, including fasting blood or plasma glucose, measured HbA1c, 75 g oral glucose tolerance testing, medical history, and self-report, where described. Screening criteria were distinguished from measurements collected as study outcomes, registry-derived information was identified explicitly, and unavailable assessment details were recorded as not reported. HBM performed the primary extraction for all 12 included studies. Each study then underwent independent second extraction and source verification by one of the six coauthors, with two included studies assigned to each coauthor; discrepancies were resolved through discussion and consensus. Every value used in the synthesis was checked against the full text or the most complete authoritative report available. Corrections were retained in the review audit trail, and the source-verified extraction was treated as the authoritative dataset for analysis and manuscript reporting.

The final source-verified dataset contained 78 eligible outcome rows across 12 studies: 72 from the original database/registry set and six from the two citation search additions. We did not create unreported statistics. Missing standard deviations were not imputed, medians were not converted to means, and absent between-group effects, confidence intervals, denominators, or paired transition counts were not reconstructed.

Outcome attribution was judged separately from simple study inclusion. A randomized CGM-specific interpretation required a contrast in which visible CGM exposure differed meaningfully between randomized groups, while relevant co-interventions were otherwise comparable. Uncontrolled and multicomponent programs were retained as supportive evidence but were not treated as proof of isolated CGM efficacy. Participant statements explicitly linking behavior to viewed CGM data were synthesized as qualitative CGM-attributed evidence. When CGM was present in both conditions and another exposure was randomized, the contrast was not interpreted as CGM versus no-CGM evidence.

### 2.6. Risk of Bias and Methodological Appraisal

Randomized intervention-effect results were assessed with Cochrane RoB 2 [8] at the outcome/result level. RoB 2 assessments were independently reviewed by Nagasravani Daruvuri and HBM. Nonrandomized intervention-effect results were assessed with ROBINS-I (2016) [9] and independently reviewed by Ramesh Raj Sunar and HBM. MMAT 2018 [10] was used for mixed methods, qualitative, or descriptive contributions that were not appropriately characterized by the two quantitative risk-of-bias tools; MMAT assessments were independently reviewed by Srinayani Guda and HBM. Disagreements were resolved through discussion and consensus. No overall MMAT percentage score was calculated. This represented a post-registration methodological adaptation to the designs encountered in the included evidence.

### 2.7. Synthesis Methods

Studies were grouped by outcome domain—dietary behavior/adherence, physical activity, psychosocial outcomes, qualitative behavioral experience, and device harms/burden—and then by evidentiary role. We distinguished direct randomized contrasts, supportive nonrandomized or multicomponent evidence, and qualitative/descriptive findings. Reported between-group estimates were used when available. Uncontrolled within-person changes were described as associations and were not converted to CGM-specific causal effects.

Meta-analysis was not performed because the studies did not form sufficiently comparable outcome-specific groups. Clinical and methodological differences included the populations studied, CGM exposure and duration, behavioral co-interventions, comparators, study designs, and outcome constructs and measurement. Pooling across these differences could have produced misleading summary estimates. Incomplete quantitative reporting imposed additional constraints. The two retrospective digital health cohorts reporting macronutrient-to-calorie changes lacked variance estimates and complete outcome-specific denominators, and overlapping participants could not be ruled out. The rice meal preference study lacked paired transition data, while the Gradiser dietary adherence result was reported only as *p* < 0.05 without effect magnitude or precision. Findings were therefore synthesized narratively; missing variances and effect estimates were not reconstructed.

Formal funnel-plot or regression-based assessment of publication bias was not performed because no outcome was meta-analyzed and the number of comparable studies per outcome was too small for meaningful assessment.

### 2.8. Certainty of Evidence

GRADE was used to rate certainty for four narrative outcome groups: dietary behavior/intake/adherence, physical activity, psychosocial well-being/anxiety/distress, and device-related harms/burden [11]. Ratings considered risk of bias, inconsistency, indirectness, imprecision, and other relevant considerations. Population-related indirectness was considered using the glycemic status ascertainment documented in Supplementary Table S2. Variation in glycemic status ascertainment was evaluated as part of population-related indirectness and was not judged sufficient to warrant an additional downgrade for the review population. Differences in biochemical confirmation, diagnostic history, self-report, and reporting completeness were nevertheless considered when assessing applicability and generalizability. HBM developed the GRADE assessments, which were independently reviewed by Srinayani Guda; disagreements were resolved through discussion and consensus. Qualitative themes were used to interpret the quantitative findings but were not assigned GRADE-CERQual ratings. Low-certainty findings are described as effects that may occur; very-low-certainty findings are presented as very uncertain.

## 3. Results

### 3.1. Study Selection

**Figure 1:**
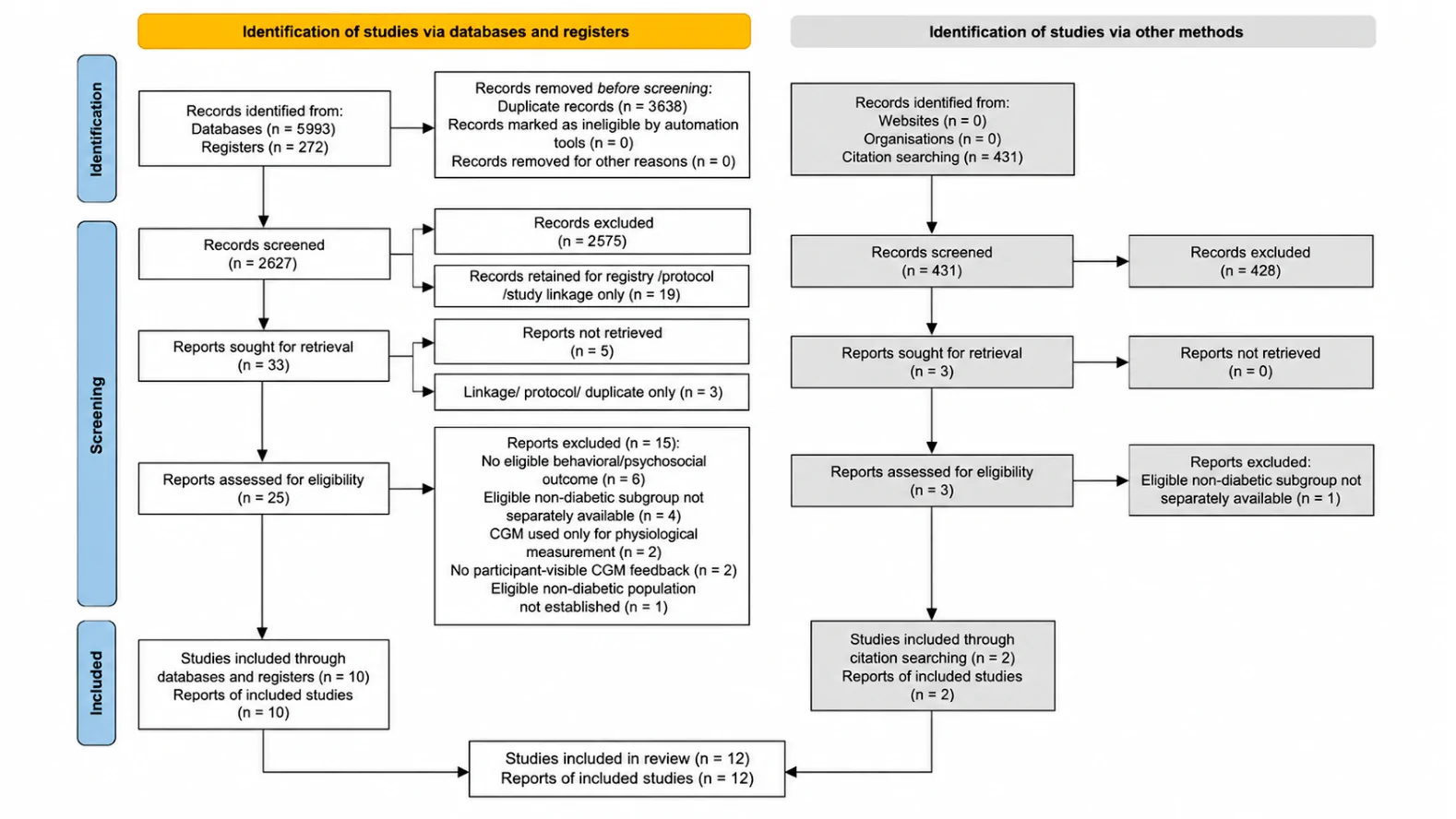
PRISMA 2020 flow diagram for database/registry searching and supplementary citation searching.

The database and registry searches yielded 6265 records: 5993 from bibliographic databases and 272 from trial registries. After 3638 duplicates were removed or suppressed, 2627 unique records were screened and 2575 were excluded at the title/abstract stage. Nineteen records were retained for registry, protocol, or study linkage only. Thirty-three reports were sought for the original effect-assessment pathway. Five had no available results report, and three retrieved records were retained only for protocol, duplicate, registry, or study linkage purposes; 25 effect reports were therefore assessed, 15 were excluded, and 10 studies entered the review from the original search. Supplementary citation searching added 431 genuinely new records for screening. Of these, 428 were excluded before report-level assessment. Three reports proceeded to eligibility assessment; one was excluded and two represented distinct eligible studies. The final review therefore included 12 reports representing 12 unique studies. Figure 1 shows the complete flow.

Across both identification pathways, 16 reports were excluded at report/full-text assessment: no eligible behavioral or psychosocial outcome (*n* = 6), an eligible non-diabetic subgroup not being separately available (*n* = 5), CGM being used only for physiological measurement (*n* = 2), absence of participant-visible CGM feedback (*n* = 2), or failure to establish an eligible non-diabetic population (*n* = 1).

### 3.2. Characteristics of Included Studies

The final evidence set comprised 12 studies published between 2019 and 2026 and included randomized trials, prospective and retrospective pre–post/cohort designs, and mixed-methods or qualitative studies [12,13,14,15,16,17,18,19,20,21,22,23]. The interventions were heterogeneous. Some trials added CGM to otherwise similar dietary or time-restricted eating support [19,20], while Gradiser et al. compared more frequent with less frequent isCGM exposure within the same structured lifestyle program [23]. Several nonrandomized studies embedded CGM in broader packages that also included nutrition education, coaching, food logging, activity monitoring or personalized digital recommendations [13,16,17,18]. Four reports contributed substantial mixed-methods, qualitative, or descriptive evidence [12,15,21,22].

CGM exposure ranged from roughly 10–14 days to several weeks, with one study using repeated intermittent exposure across 12 weeks. FreeStyle Libre systems predominated, sometimes paired with commercial or study-specific digital platforms. Participants were adults without diagnosed diabetes or separately reported normoglycemic subgroups, but they were clinically diverse: university students, trained athletes, adults with overweight or obesity, people at elevated risk of type 2 diabetes, and self-selected digital health users. In study-specific descriptions, terms such as “healthy” and “normoglycemic” reflect the classifications used in the original reports rather than a uniform assessment of metabolic health across all included participants. Table 1 summarizes the studies and their role in the synthesis. Study-specific methods for assessing diabetes and glucose tolerance status, including reported biochemical criteria, history-based or self-reported classifications, and unavailable assessment details, are provided in Supplementary Table S2.

Explicit normal-range biochemical eligibility or subgroup criteria were reported in three studies: fasting blood glucose < 5.6 mmol/L in the two Chekima studies [20,22] and HbA1c < 5.7% in the extracted Torii-Goto subgroup [16]. These were test-specific criteria rather than a uniform confirmation of normal glucose tolerance across studies. Whelan et al. excluded self-reported diabetes and HbA1c ≥ 6.5%; three participants had HbA1c values of 6.0–6.4% according to the source report [12]. The registry linked to Gradiser et al. described blood glucose and HbA1c screening to exclude prediabetes/diabetes, but numerical thresholds and confirmation of the screening procedure in the published abstract were not available (NCT07026903; Supplementary Table S2). In the remaining seven studies, objective confirmation of normal glycemia was not documented in the available reports; population descriptions instead relied on diagnostic history, self-report, disease-exclusion criteria, or insufficiently described assessment [13,14,15,17,18,19,21]. These populations were therefore not assumed to be uniformly normoglycemic.

### 3.3. Dietary Behavior and Adherence

Eight studies contributed quantitative dietary or adherence outcomes. The strongest direct evidence still came from two randomized comparisons in which visible CGM differed from the relevant comparator [19,20]. In the 8-week Chekima trial, both groups received low-GI/GL nutrition education and the intervention group additionally received participant-visible rtCGM. Compared with education alone, the CGM group had greater reductions in carbohydrate intake (−15.2 g; 95% CI −20.9 to −9.5; *p* = 0.04), carbohydrate percentage of energy (−3.6 percentage points; 95% CI −6.9 to −0.3; *p* = 0.03), glycemic index (−10.0; 95% CI −13.1 to −6.9; *p* = 0.006), and glycemic load (−19.9; 95% CI −29.0 to −10.7; *p* = 0.008). Total energy did not show a clear between-group difference (−17 kcal; 95% CI −53 to 17; *p* = 0.32) [20].

The 14-day time-restricted eating pilot randomized 22 participants. Two participants assigned to TRE + CGM were excluded after randomization for medical reasons, leaving 9 in the analyzed CGM group and 11 in TRE-only. Mean adherent days were 10.0 versus 8.6, and 5/9 versus 3/11 participants maintained the 8 h eating window on at least 80% of days. The abstract did not report dispersion for adherent days or a formal comparative effect estimate, so the size and precision of any CGM contribution remain uncertain [19].

The two studies identified through citation searching added useful but more qualified dietary evidence. In Gradiser et al., 35 women with overweight or obesity were randomized to the same 12-week exercise and dietary-counseling program; isCGM was used at baseline, midpoint, and study end in the intervention group and at baseline and study end in controls. Dietary adherence was reported as better with the more frequent isCGM schedule (*p* < 0.05), but the adherence instrument, numerical effect, variance, confidence interval, and analyzed denominator were not reported [23]. The study therefore informs exposure frequency rather than a simple CGM-versus-no-CGM comparison. In the rice meal study by Chekima et al., meal order was randomized, but the behavioral comparison was a within-person pre/post assessment after personalized rtCGM feedback and low-GI/GL education. Preference shifted from the white rice meal in 9/14 participants (64.3%) before feedback to the parboiled basmati rice meal in 10/14 (71.4%) afterward; the overall paired preference distribution changed significantly (*p* = 0.012) [22]. Because transition cells were not reported and CGM feedback was bundled with explanation and education, this finding was treated as supportive rather than randomized CGM efficacy.

The remaining nonrandomized studies generally moved in a behaviorally favorable direction but were embedded in multicomponent programs. In the HbA1c-defined subgroup of Torii-Goto et al. (HbA1c < 5.7%), a composite lifestyle-consciousness VAS increased from 492 to 566 (*p* < 0.01) after educator feedback and two months of diet/exercise self-management [16]. Park reported an increase in dietary self-efficacy from 6.84 (SD 1.64) to 7.69 (SD 1.26; *p* < 0.05), whereas the overall Nutrition Quotient changed little (43.74 [SD 6.32] to 44.59 [SD 6.24]; *p* = 0.485) [18]. Two retrospective digital health cohorts also reported favorable macronutrient shifts in subgroups labeled as healthy or normoglycemic in the source reports without documented biochemical verification of those classifications. Variance and some outcome-specific denominators were unavailable, and the cohorts may not have been fully independent [13,17]. These estimates were retained as supportive narrative evidence only.

### 3.4. Physical Activity

Five studies contained quantitative physical activity information, but the only directly randomized CGM-specific estimate came from Chekima et al. Participants were asked not to increase physical activity during the dietary intervention, and activity was assessed with the IPAQ Short Form. The between-group difference in change was −28.5 MET-min/week (95% CI −171.9 to 114.8; *p* = 0.69), providing no clear evidence that CGM feedback either increased or decreased activity [20].

Supportive evidence was inconsistent and methodologically weaker. In the Season of Me cohort, adjusted self-logged activity increased from about 50 to 109 min/day in users classified as healthy in the source report; separately, objectively derived time with heart rate > 110 bpm increased by 2.9 min/day (*p* = 0.009; outcome-specific *n* = 764) [13]. The primary report gives *n* = 746 for the source-labeled normoglycemic cohort but *n* = 764 for this activity analysis, so the outcome-specific denominator was retained as reported. Park’s exercise-frequency score increased in the multicomponent pre–post program, but the corresponding *p* value was 0.512 [18]. In the randomized SIGNAL feasibility study, 13/45 participants changed at least one physical activity goal, yet no clean randomized comparison isolated CGM feedback, and no clear trend in objective activity was reported over six weeks [12].

### 3.5. Psychosocial Outcomes

No study supplied a robust, extractable numerical between-group estimate of a CGM-attributable psychosocial effect. In the 12-week Walson randomized trial, emotional well-being, PHQ-9, GAD-7, and PSQI outcomes improved over time in both CGM and glucometer groups, but the conference abstract reported no between-group differences and gave no numerical between-group values [14]. The TRE pilot likewise reported no consistent adverse psychological pattern on WHO-5 or DASS-21 measures without numerical results [19].

Pujol-Busquets et al. reported numerical WHO-5 and EQ-5D changes across low-carbohydrate/high-fat and high-carbohydrate/low-fat phases [15]. We did not interpret those quantitative differences as CGM effects because participants used CGM in both diet conditions and were not instructed to modify behavior from the readings. The contrast therefore reflects diet phase differences during CGM use rather than CGM versus no-CGM psychosocial effects.

### 3.6. Qualitative Behavioral and Psychosocial Experience

Qualitative and mixed-methods findings help explain why the quantitative results were inconsistent. Across five studies, participants described greater awareness of links between meals, activity, and glucose; learning from real-time patterns; motivation; empowerment; self-regulation; and a sense of accountability [12,15,19,21,22]. In the TRE pilot, participants described CGM as reassuring during fasting and as a source of accountability [19]. Pujol-Busquets et al. identified themes of empowerment and learning, usability and practicality, and enhancement and interest; participants felt that real-time data could reinforce dietary awareness and self-efficacy even though they had not been instructed to change their behavior based on the readings [15].

The two smallest behavioral studies also showed that feedback did not translate uniformly into action. In the Chekima rice meal study, most participants attributed their change in rice preference to the postprandial glucose patterns they had seen, although the study did not report counts, quotations, or a formal qualitative analytic method [22]. At three months, 12 participants responded to follow-up: 33.3% reported selecting lower-GI brown or basmati rice, 41.7% reported switching between lower- and higher-GI varieties, and 25% said changing rice variety was difficult; numerators were not reported and were not inferred. In Liang’s six-person pilot, several participants changed meal timing, ate earlier, avoided snacks, or reduced late-night sweets, whereas others ignored glucose spikes because they felt well or deferred making changes until later [21].

Negative or mixed experiences were recurrent as well. Participants described uncertainty about interpreting glucose information, forgetting to scan, problems with adhesion and durability, frustration with device requirements, and self-consciousness about visible sensors [12,15]. These observations are mechanistically important: if a sensor is difficult to wear, remember, or interpret, the feedback loop itself may weaken even when glucose measurement is technically adequate.

### 3.7. Device-Related Harms and Burden

Four studies contributed descriptive evidence on device-related harms or burden [12,15,19,21]. In SIGNAL, 41 sensors were declared faulty or displaced, and only 22/45 participants completed the intervention using the minimum required number of Libre sensors; some participants described irritation and self-consciousness, although event frequencies were not reported [12]. Pujol-Busquets et al. described frequent scanning, adhesion, calibration/durability, and data interpretation difficulties without reliable event counts [15]. Liang reported no skin irritation or pain among six participants [21]. The TRE abstract reported no consistent adverse psychological pattern but provided no numerical WHO-5/DASS-21 results [19]. These descriptive, non-comparative findings provide signals for further investigation rather than reliable estimates of event frequency or comparative risk. The certainty of this evidence was very low, and these reports do not establish that CGM causes psychological harm.

### 3.8. Risk of Bias and Methodological Appraisal Results

RoB 2 was applied to six randomized result assessments. Overall risk was high for Chekima’s glycemic load result, both Tater outcomes, Walson’s emotional well-being result, and Gradiser’s dietary adherence result. Chekima’s IPAQ physical activity result was judged as having some concerns overall. The recurring issues were incomplete reporting of randomization or prespecification, post-randomization exclusions or missing outcome information, subjective/self-reported outcome measurement, and limited reporting of the planned analysis. Gradiser’s high overall judgment reflected the abstract/registry evidence base, especially missing attrition and numerical adherence detail [14,19,20,23].

**Figure 2:**
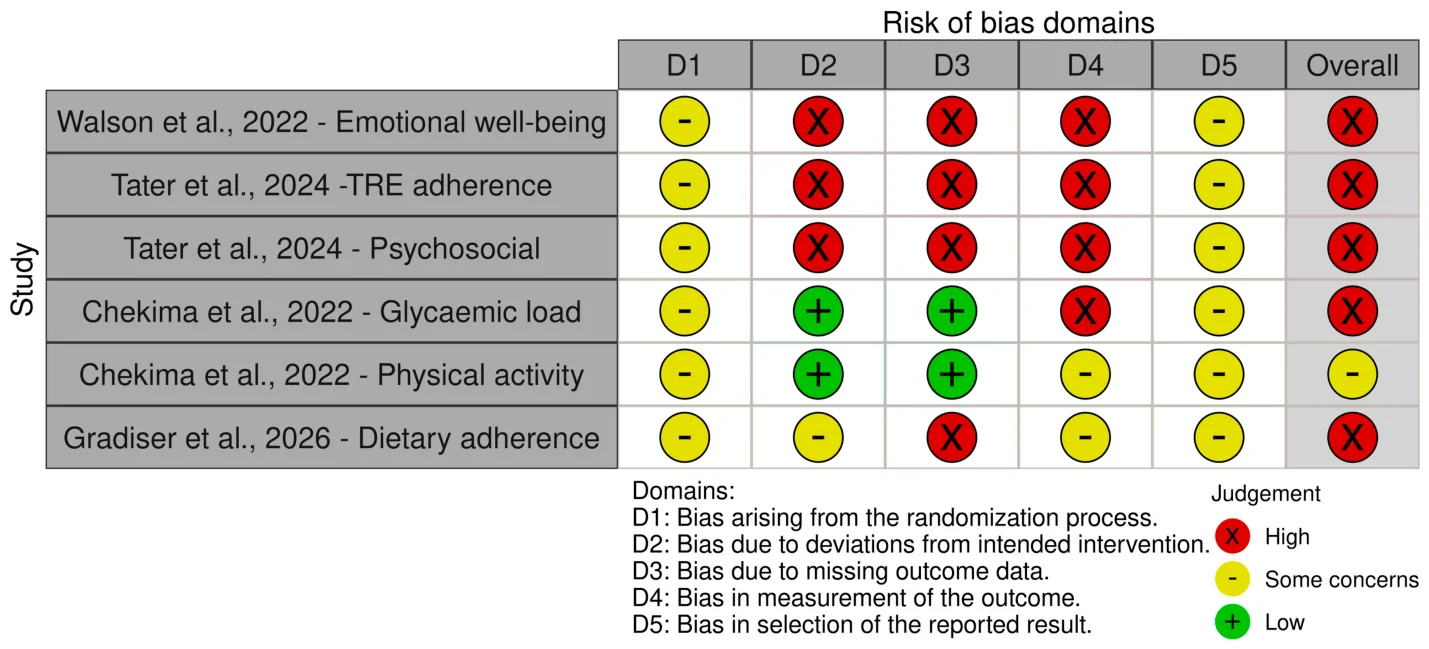
Outcome-specific RoB 2 traffic light plot for randomized intervention-effect results [14,19,20,23].

**Figure 3:**
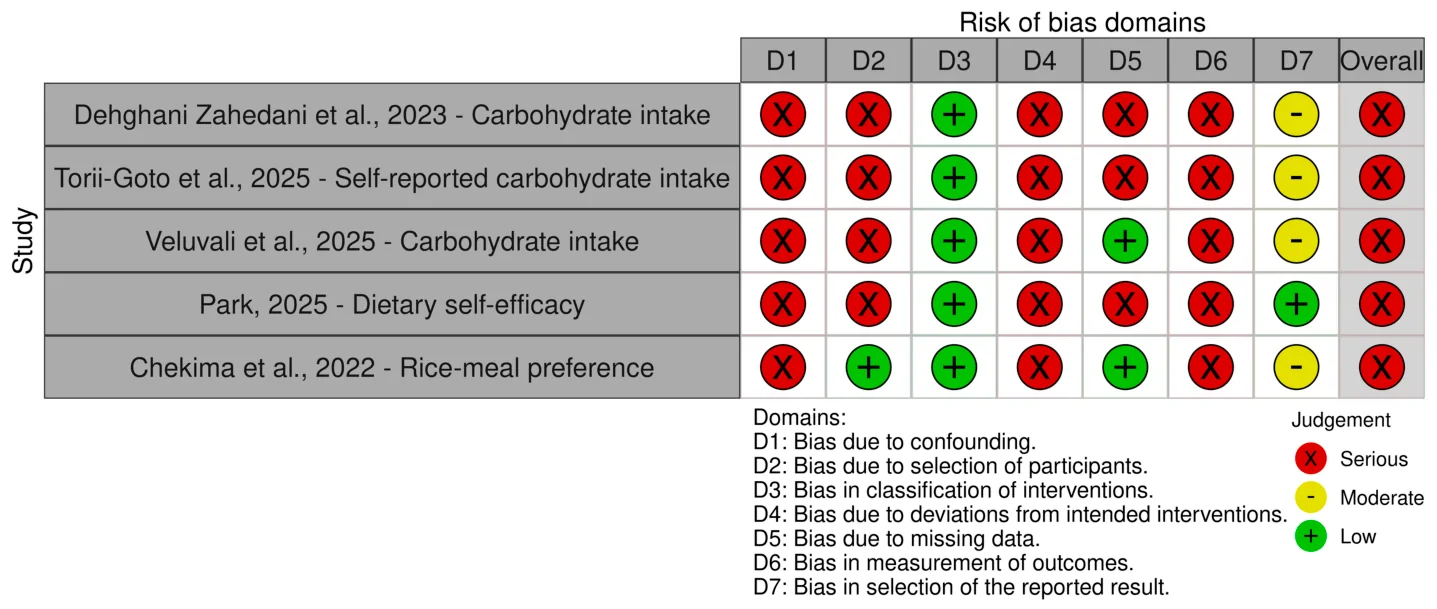
ROBINS-I traffic light plot for nonrandomized intervention-effect results [13,16,17,18,22].

All five nonrandomized intervention-effect appraisals—Dehghani Zahedani, Torii-Goto, Veluvali, Park, and the behavioral pre/post result from the Chekima rice meal study—were judged at overall serious risk of bias with ROBINS-I [13,16,17,18,22]. Recurrent concerns included confounding, uncontrolled pre–post or retrospective designs, intervention co-components, self-reported outcomes, and selection into analysis. Four studies were also appraised with MMAT for mixed-methods, qualitative, or descriptive contributions [12,15,21,22]. MMAT criteria were reported individually rather than converted to an overall percentage score. Detailed judgments are provided in the Supplementary Tables and Figure 2 and Figure 3.

### 3.9. Certainty of Evidence Results

GRADE ratings are summarized in Table 2. The direct dietary behavior/intake/adherence body was rated low certainty because of serious risk-of-bias limitations and serious imprecision. The additional Gradiser randomized exposure frequency result and the Chekima within-person preference findings were considered supportive but did not replace the direct CGM-versus-relevant-comparator body. Physical activity was rated very low certainty because it rested on one small randomized trial with a wide confidence interval. Psychosocial effects were also very low certainty: the two randomized reports were small, high risk, and lacked usable numerical between-group estimates. Device harms/burden were very low certainty because ascertainment was largely descriptive or self-reported and event frequencies and comparative estimates were usually absent.

Glycemic status ascertainment varied across the outcome-specific evidence bodies. The principal numerical dietary and physical activity estimates came from the Chekima trial, which required fasting blood glucose < 5.6 mmol/L [20], whereas the two randomized psychosocial reports did not adequately describe glycemic status ascertainment [14,19]. Device burden evidence also came from populations with differing or incompletely characterized glycemic status (Supplementary Table S2). No additional downgrade for population-related indirectness was applied for the review population; the rationale is provided in Supplementary Table S6A, footnote i. The certainty ratings do not establish that the same effects apply specifically to adults with uniformly, objectively confirmed normal glucose tolerance.

### 3.10. Quantitative Synthesis Feasibility

No meta-analysis was performed because the available studies did not provide sufficiently comparable outcome-specific groups. Dietary evidence included CGM added to education or time-restricted eating, different frequencies of CGM exposure, and uncontrolled multicomponent programs, which addressed distinct intervention contrasts. Direct CGM-specific comparisons within the physical activity and psychosocial domains were too sparse or incompletely reported to support pooling. Incomplete effect and variance reporting, and unresolved overlap between the retrospective digital health cohorts imposed further constraints. Findings were therefore synthesized narratively by outcome domain and evidentiary role rather than combined into potentially misleading pooled estimates.

## 4. Discussion

### 4.1. Principal Findings

The clearest signal was dietary, with low-certainty evidence. The Chekima trial showed greater reductions in selected carbohydrate-related measures with CGM plus low-GI/GL education than with education alone [20]. The TRE pilot reported numerically greater adherence but provided insufficient information to estimate precision [19]. Additional supportive findings came from the CGM exposure frequency trial [23] and the nonrandomized behavioral comparison in the rice meal study [22].

Physical activity evidence is much less informative. The only randomized estimate was close to null and imprecise, whereas increases reported in uncontrolled digital health programs occurred within packages that also included activity monitoring, individualized recommendations, and other behavior change components. Those cohort findings cannot be used to confirm that glucose feedback itself increases exercise.

Psychosocial effects remain a major gap. Randomized reports relevant to well-being, depression, anxiety, stress, or sleep did not provide usable between-group numerical estimates [14,19]. Qualitative work described empowerment, curiosity, learning, and reassurance, but also frustration, uncertainty, scanning burden, and self-consciousness. Those experiences can coexist. Real-time biometric feedback may be engaging for one user and burdensome for another, depending on expectations, interpretation, usability, and the quality of guidance around the data.

### 4.2. Why Attribution Matters

Interpretation depended on the comparison each study actually made. We distinguished randomized CGM-specific contrasts, associations within multicomponent or uncontrolled programs, and participant-reported experiences. More frequent versus less frequent CGM use informs exposure frequency, not use versus no use [23]. Likewise, nonrandomized changes after combined CGM feedback and education cannot isolate the contribution of glucose data [22]. Keeping these evidence types separate avoids treating all findings as evidence of independent CGM efficacy.

### 4.3. Relationship to Previous Reviews

Earlier reviews of CGM in people without diabetes emphasized possible roles in wellness, personalized nutrition, and exercise while repeatedly calling for stronger evidence [1,2]. Oganesova et al. later highlighted inconsistent support for consumer health claims and raised device acceptability and possible adverse eating effects as areas of concern [3]. Our findings are consistent with that caution. Dietary behavior carries the clearest signal, but certainty remains low; psychosocial safety and benefit are still too sparsely measured for confident conclusions.

Liao et al. reviewed a broader set of 23 studies in non-diabetic populations, including glycemic outcomes, body weight, dietary behavior, adherence, and measurement performance [4]. Their question is wider than ours. We excluded clinically managed prediabetes, required participant-visible feedback for the principal behavioral causal question, and separated physiological measurement-only use and non-attributable psychosocial contrasts from CGM-effect evidence. The more cautious conclusion here, therefore, does not conflict with evidence that lifestyle programs containing CGM can improve short-term behavior or metabolic measures; it addresses a narrower question—how much of the observed behavioral or psychosocial effect can currently be assigned to seeing CGM feedback itself?

### 4.4. Clinical, Consumer, and Research Implications

For adults without diabetes, the most favorable dietary findings were observed when participant-visible CGM was combined with nutritional education or structured lifestyle interventions [19,20,22,23]. The randomized comparison of CGM plus low-GI/GL education versus education alone suggests a possible incremental benefit in that setting, but does not establish the effectiveness of CGM without accompanying education [20]. The low-certainty dietary evidence therefore supports a potential role for CGM as an adjunctive behavioral biofeedback tool within structured support, rather than as an independently validated method for broad or durable lifestyle improvement.

Psychosocial safety remains unresolved: the available evidence neither establishes that participant-visible CGM causes psychological harm nor adequately rules such harm out. Although the randomized reports included well-being, mood, or anxiety measures, they lacked usable numerical between-group estimates of CGM-attributable psychosocial effects [14,19]. Their limited findings should therefore not be interpreted as evidence of psychological safety. Future studies should prospectively assess anxiety related to glucose excursions, excessive dietary restriction, food-related distress, disordered eating behaviors, and compulsive interpretation of glucose data, alongside quality of life and device-related distress, using validated instruments appropriate to the constructs assessed. These assessments are particularly important when CGM is marketed directly to people without diabetes.

Future randomized trials should isolate visible CGM feedback from co-interventions whenever possible, prespecify behavioral and psychosocial outcomes, keep randomized participants in clearly described analyses, and report between-group estimates with confidence intervals. Objective activity measures and validated dietary instruments should complement self-report. Longer follow-up is needed to determine whether early curiosity becomes sustained behavior change or fades as novelty diminishes. Sensor failures, adhesion problems, scanning burden, irritation, self-consciousness, interpretation difficulties, and discontinuation should be assessed prospectively using consistent definitions, with denominators and follow-up duration reported explicitly. Appropriate comparator groups are needed to estimate comparative risk.

### 4.5. Strengths and Limitations of This Review

Several features strengthen this review. It was prospectively registered; it searched seven bibliographic databases and two international registries; it deliberately omitted behavioral outcome terms from the search strategy to reduce the chance of missing secondary or qualitative outcomes; and it supplemented the original search with iterative backward and forward citation searching. Every synthesis value was checked against the source report, and qualitative and device burden evidence were retained rather than discarded. Most importantly, the analysis separated randomized CGM-specific contrasts from uncontrolled or multicomponent programs and from participant-reported experience. Narrative synthesis avoided combining clinically and methodologically distinct comparisons into potentially misleading pooled estimates; incomplete quantitative reporting and unresolved cohort overlap provided additional reasons not to pool.

The main limitations come from the evidence base. Only 12 studies met the focused criteria, and direct randomized evidence remained sparse. Two randomized sources were conference abstracts with limited reporting, and one additional randomized study was available only through its published abstract and a linked registry record. Several studies were short, small, uncontrolled, or reliant on self-report. Glycemic status ascertainment was heterogeneous: some studies used explicit fasting glucose or HbA1c criteria, whereas others relied on diagnostic history, self-report, or incompletely described assessment. Consequently, the findings cannot be assumed to apply uniformly to adults with objectively confirmed normal glucose tolerance, and whether behavioral or psychosocial responses differed according to baseline glycemic status could not be determined from the available reporting. The rice meal preference study lacked paired transition data, limiting quantitative synthesis of that outcome. Outcome-specific denominators were sometimes missing, and two related digital health cohorts may not be fully independent. At the review-process level, primary screening and extraction were centralized in one reviewer before independent second assessment and source verification, and some eligibility or appraisal decisions necessarily relied on the most complete available report when complete full-text reporting was unavailable. Restricting the database searches to records from 2015 onward may also have omitted relevant earlier behavioral or psychosocial evidence.

## 5. Conclusions

Participant-visible CGM may influence selected dietary behaviors and adherence in adults without diabetes, especially when it is embedded in structured education or lifestyle support. The evidence does not yet establish a large, durable, or independent behavior change effect. Randomized evidence for physical activity is very uncertain. Psychosocial benefit and safety remain unresolved; the absence of demonstrated harm should not be interpreted as evidence of safety. Qualitative studies help explain the technology’s appeal—users can learn from glucose patterns, feel accountable, and reconsider food choices or meal timing—while also documenting practical and interpretive burdens. Until larger, better-reported trials isolate the contribution of visible glucose feedback, CGM in adults without diabetes is best viewed as a potentially engaging adjunctive biofeedback tool with uncertain independent benefit and incompletely characterized psychosocial consequences.

## Figures and Tables

**Table 1:** Characteristics of included studies and role in synthesis.

Study	Country/Design	Eligible Population (N)	Participant-Visible CGM/Co-Intervention	Comparator	Primary Contribution to This Review
Whelan et al., 2019 [12]	United Kingdom; 3-arm randomized feasibility trial + interviews	Adults ≥ 40 years at moderate/high T2D risk; self-reported diabetes and HbA1c ≥ 6.5% excluded; three participants had HbA1c 6.0–6.4% (45)	Libre feedback sequenced with Fitbit feedback; no prescribed behavior change	Randomized feedback schedules	Feasibility/qualitative behavior; device burden; not a clean randomized CGM-specific behavior-effect estimate
Dehghani Zahedani et al., 2023 [13]	Remote real-world retrospective cohort	Source-labeled “normoglycemic” subgroup; biochemical verification not reported (cohort description 746; outcome-specific Ns varied)	Season of Me: visible Libre + activity/heart-rate tracking + food logs + personalized digital recommendations	Within-person baseline vs. program	Supportive dietary/activity evidence; multicomponent, uncontrolled
Walson et al., 2022 [14]	Setting not reported; 12-week parallel randomized trial (conference abstract)	Non-diabetic wellness participants (25; 14 CGM vs. 11 control)	CGM + Levels within low-carbohydrate wellness program	Same program + glucometer	Direct randomized psychosocial evidence; numeric between-group values unavailable
Pujol-Busquets et al., 2026 [15]	United States free-living; randomized counterbalanced crossover mixed-methods	Trained non-diabetic men aged 30–50 years (10)	Libre 2 + Levels during both 31-day diet phases	LCHF vs. HCLF; CGM in both phases	Qualitative awareness/empowerment/burden; diet phase WHO-5/EQ-5D not CGM-attributable
Torii-Goto et al., 2025 [16]	Japan; prospective pre–post intervention	Separately reported HbA1c-defined subgroup, HbA1c < 5.7% (18)	isCGM assessment + individualized educator feedback + 2-month self-management	Within-person pre–post; no CGM-free control	Supportive lifestyle-awareness evidence; multicomponent, uncontrolled
Veluvali et al., 2025 [17]	United States remote users; retrospective cohort	Self-reported “healthy” subgroup; biochemical verification not reported (785)	January V2: visible CGM/heart-rate tracking + food logging + personalized digital feedback	Within-participant program period	Supportive dietary evidence; variance/outcome-specific N unavailable
Park, 2025 [18]	South Korea; single-arm pre–post	University students without diabetes (27 analyzed; 40 consented)	4-week Libre + group education + weekly coaching + apps/weight monitoring	Within-person pre–post	Supportive dietary self-efficacy/NQ evidence; multicomponent, uncontrolled
Tater et al., 2024 [19]	New Zealand; 2-arm randomized pilot (conference abstract)	Adults described as “healthy”; glycemic screening unspecified in the abstract (22 randomized; 20 analyzed)	14-day 16:8 TRE + visible CGM	TRE only	Direct randomized TRE adherence and psychosocial evidence; incomplete statistics
Chekima et al., 2022 [20]	Malaysia; parallel RCT	Non-diabetic overweight/obese adults aged 18–35 years (40)	Low-GI/GL education + visible Libre	Same education without CGM	Direct randomized dietary and physical activity effects
Liang, 2024 [21]	Japan; single-arm mixed-methods pilot	Graduate students without diagnosed chronic disease (6)	~1 month visible Libre without personalized recommendations	No concurrent control	Qualitative meal-timing/food-choice responses; exploratory device experience
Chekima et al., 2022 [22]	Malaysia; randomized crossover meal study with nonrandomized within-person behavioral pre/post assessment	Adults aged 18–35 years with BMI ≥ 25 kg/m^2^ and normal fasting glucose; diabetes excluded (14 analyzed)	Visible FreeStyle Libre during rice meal testing; personalized trendlines explained with low-GI/GL education	Within-person meal preference before vs. after feedback/education; no randomized CGM/no-CGM behavioral comparator	Supportive meal-preference and 3-month food-selection evidence; qualitative CGM-attributed reasoning
Gradiser et al., 2026 [23]	Croatia; 12-week two-group randomized controlled trial	Women aged >18 years with BMI > 25 kg/m^2^; prediabetes/diabetes excluded according to the linked registry (35 randomized; 17 intervention, 18 control)	Structured lifestyle program + isCGM at baseline, midpoint, and study end	Same lifestyle program + isCGM at baseline and study end	Randomized dietary adherence evidence for greater vs. lower isCGM exposure; *p* < 0.05 only, magnitude/precision not reported

Population labels are reported with their ascertainment limitations. A stated normal fasting glucose or HbA1c criterion should not be interpreted as uniform confirmation of normal glucose tolerance across all studies. Detailed assessment methods and reporting limitations are provided in Supplementary Table S2.

**Table 2:** Summary of findings and GRADE certainty.

Outcome	Evidence Body	Key Finding	Main Downgrading Considerations	Certainty
Dietary behavior/intake/adherence	2 randomized trials (direct body); additional supportive randomized exposure frequency and nonrandomized evidence	Visible CGM may improve selected dietary behaviors or adherence, but the magnitude and independent contribution remain uncertain.	Serious risk of bias; serious imprecision	Low
Physical activity	1 randomized trial	No clear between-group difference: −28.5 MET-min/week (95% CI −171.9 to 114.8).	Serious risk-of-bias concerns at body level; very serious imprecision	Very Low
Psychosocial well-being/anxiety/distress	2 randomized reports	Direction-only reports; no usable numerical between-group effect estimate.	Very serious risk of bias; very serious imprecision	Very Low
Device-related harms/burden	4 studies; descriptive/non-comparative harms evidence	Practical and psychological burdens were reported, but frequency and comparative risk are uncertain.	Serious ascertainment/reporting limitations; very serious imprecision	Very Low

## Data Availability

No new primary data were generated or collected for this systematic review. All evidence synthesized was derived from previously reported studies cited in the manuscript. Source-verified study characteristics and outcome extractions, complete search strategies and citation search logs, risk-of-bias and methodological appraisal materials, and GRADE evidence profiles are provided in the Supplementary Materials.

## Supplementary Materials

The following supporting information can be downloaded at: https://www.mdpi.com/article/10.3390/medicina62091814/s1, Supplementary File S1: Search strategies and operational log; Table S1: Full-text/available-report exclusions; Table S2: Source-verified study characteristics and outcome extraction; Table S3: RoB 2 assessments; Table S4: ROBINS-I assessments; Table S5: MMAT 2018 assessments; Table S6A: GRADE evidence profile; Table S6B: GRADE summary of findings. A completed PRISMA 2020 checklist is also provided with the submission.
