# Supplementary material for: Behavioral and Psychosocial Effects of Participant-Visible Continuous Glucose Monitoring in Adults Without Diabetes: A Systematic Review
Source: Medicina (Kaunas). 2026 Sep 20;62(9):1814. doi: 10.3390/medicina62091814 (PMC13609687; doi:10.3390/medicina62091814)
Supplement: Supplementary file 1 [file medicina-62-01814-s001.zip › Supplementary_Table_S6A_GRADE_Evidence_Profile_Medicina.pdf]

Supplementary Table S6A. GRADE Evidence Profile

Review authors: Hima Bindu Makkena et al.

Question: Participant-visible continuous glucose monitoring (CGM) in free-living settings compared with no CGM feedback, baseline status, or standard lifestyle intervention without CGM feedback in adults without diabetes

Setting: Free-living / real-world settings

Bibliography: Included studies are cited in manuscript references 12–23 and detailed in Supplementary Table S2.

Outcome	No. of studies	Study design	Risk of bias	Inconsistency	Indirectness	Imprecision	Other considerations	Impact	Certainty	Importance
Dietary behavior, dietary intake and adherence	2	Randomized trials	Serious[a]	Not serious	Not serious[l]	Serious[b]	None	Two small randomized trials suggested that participant-visible CGM may improve some dietary behaviors. In an 8-week RCT of 40 overweight/obese non-diabetic young adults, rtCGM added to low-GI/GL education produced greater reductions in carbohydrate intake, carbohydrate percentage of energy, glycemic index and glycemic load than education alone, while total energy intake showed no clear between-group difference. In a 14-day TRE pilot RCT, participants receiving CGM averaged 10.0 adherent days versus 8.6 days with TRE alone, and 5/9 versus 3/11 met the prespecified adherence criterion; however, comparative statistical estimates were not reported. Overall, participant-visible CGM may improve selected dietary behaviors, but confidence in the magnitude of the effect is limited.	LOW	CRITICAL

Outcome	No. of studies	Study design	Risk of bias	Inconsistency	Indirectness	Imprecision	Other considerations	Impact	Certainty	Importance
								Additional supportive evidence was identified through citation searching. Chekima et al. (2022; rice-meal preference study) reported within-person and descriptive changes in meal preference after exposure to participant-visible CGM, but the CGM effect itself was not randomized. Gradiser et al. (2026) reported improved dietary adherence with more frequent isCGM exposure compared with less frequent isCGM exposure (P<0.05), but no numerical effect estimate or confidence interval was available. These studies were therefore considered supportive rather than additional direct CGM-versus-no-CGM evidence.		
Physical activity behavior	1	Randomized trial	Serious[c]	Not serious	Not serious[l]	Very serious[d]	None	One 8-week randomized trial in 40 overweight/obese non-diabetic young adults found no clear difference in physical activity between rtCGM plus low-GI/GL education and education alone. The between-group difference in IPAQ physical activity was -28.5 MET-min/week (95% CI -171.9 to 114.8; p=0.69). The effect of participant-visible CGM on physical activity therefore remains very uncertain.	VERY LOW	CRITICAL
Psychosocial well-being/anxiety/distress	2	Randomized trials	Very serious[e]	Not serious	Not serious[l]	Very serious[f]	None	Two small randomized studies provided very limited evidence on psychosocial	VERY LOW	CRITICAL

Outcome	No. of studies	Study design	Risk of bias	Inconsistency	Indirectness	Imprecision	Other considerations	Impact	Certainty	Importance
								effects. One reported improvements over time in emotional well-being, depression, anxiety and sleep in both CGM and comparator groups, with no reported between-group differences, but numerical results were unavailable. The second reported no consistent adverse psychological effects on WHO-5 or DASS-21 measures, but also provided no numerical values. The effect of participant-visible CGM on psychosocial well-being, anxiety or distress therefore remains very uncertain.		
Device-related harms and burden	4	Non-randomized/descriptive contributions	Serious[g]	Not serious	Not serious[i]	Very serious[h]	None	Four studies with mixed parent designs contributed descriptive, non-comparative evidence on device-related harms and burden. Reported problems included sensor adhesion or displacement, scanning requirements, durability concerns, irritation, self-consciousness and other device-related inconvenience. Whelan et al. (2019) reported 41 faulty or displaced sensors, and only 22/45 participants completed the intervention using the minimum required number of sensors. Pujol-Busquets et al. (2026) described scanning, adhesion and durability difficulties without event counts.	VERY LOW	CRITICAL

Outcome	No. of studies	Study design	Risk of bias	Inconsistency	Indirectness	Imprecision	Other considerations	Impact	Certainty	Importance
								**Liang (2024)** reported no skin irritation or pain among six participants. Tater et al. (2024) reported no consistent adverse psychological pattern, but numerical psychosocial data were unavailable. Device-related practical and psychological burdens were reported during participant-visible CGM use, but the frequency, comparative risk and magnitude of these harms remain very uncertain.		

Explanations

a. Downgraded one level because both randomized trials had important methodological limitations. The larger dietary trial was open-label and relied on self-reported dietary outcomes, with additional concerns regarding randomization and selective reporting, while the smaller TRE adherence trial had post-randomization exclusions, missing outcome data, and incomplete statistical reporting.

b. Downgraded one level because the total randomized evidence was limited and the smaller TRE adherence trial did not report confidence intervals or a comparative statistical test; however, the larger dietary trial provided confidence intervals for several direct dietary outcomes.

c. Downgraded one level for risk of bias because the single randomized trial had overall some concerns, including concerns related to randomization, self-reported physical activity measurement, and selection of the reported result.

d. Downgraded two levels because evidence came from a single small randomized trial and the confidence interval was wide, including both potentially lower and higher physical activity with CGM.

e. Downgraded two levels because both randomized studies were judged at overall high risk of bias. Important limitations included incomplete outcome reporting, post-randomization exclusions, lack of blinding and reliance on self-reported psychosocial outcomes.

f. Downgraded two levels because both studies were small and neither provided sufficient numerical between-group effect estimates, confidence intervals or dispersion data to estimate the magnitude or precision of the psychosocial effect.

g. Downgraded one level because harms and device-burden outcomes were generally collected descriptively or through participant self-report, without standardized ascertainment or blinded assessment. Several reports did not provide frequencies or validated burden measures.

h. Downgraded two levels because the evidence was sparse, event counts were frequently absent, sample sizes were small in several studies, and no reliable comparative harm estimate with confidence intervals could be calculated.

i. Glycemic-status ascertainment varied across studies, ranging from explicit fasting-glucose or HbA1c criteria to diagnostic history, self-report, or incompletely reported assessment. This variation was considered in relation to the review population and was not judged sufficient to warrant an additional downgrade for indirectness. Generalizability to adults with uniformly, objectively confirmed normal glucose tolerance nevertheless remains limited.
